# Inverse problem for multi-body interaction of nonlinear waves

**DOI:** 10.1038/s41598-017-03163-4

**Published:** 2017-06-14

**Authors:** Alessia Marruzzo, Payal Tyagi, Fabrizio Antenucci, Andrea Pagnani, Luca Leuzzi

**Affiliations:** 1 NANOTEC-CNR, Institute of Nanotechnology, Soft and Living Matter Laboratory, Rome, Piazzale A. Moro 2, I-00185 Roma, Italy; 2 Department of Applied Science and Technology, Politecnico di Torino, 10129 Torino, Italy; 3 Human Genetics Foundation, Molecular Biotechnology Center, 10126 Torino, Italy; 4 Dipartimento di Fisica, Università di Roma “Sapienza”, Piazzale A. Moro 2, I-00185 Roma, Italy

## Abstract

The inverse problem is studied in multi-body systems with nonlinear dynamics representing, e.g., phase-locked wave systems, standard multimode and random lasers. Using a general model for four-body interacting complex-valued variables we test two methods based on pseudolikelihood, respectively with regularization and with decimation, to determine the coupling constants from sets of measured configurations. We test statistical inference predictions for increasing number of sampled configurations and for an externally tunable *temperature*-like parameter mimicing real data noise and helping minimization procedures. Analyzed models with phasors and rotors are generalizations of problems of real-valued spherical problems (e.g., density fluctuations), discrete spins (Ising and vectorial Potts) or finite number of states (standard Potts): inference methods presented here can, then, be straightforward applied to a large class of inverse problems. The high versatility of the exposed techniques also concerns the number of expected interactions: results are presented for different graph topologies, ranging from sparse to dense graphs.

## Introduction

Multi-body inference turns out to be essential whenever non-linear response is crucial for a system properties. Light mode interaction in ultra-fast multimode lasers^1–6^, random lasers^7–10^, multi-variable clause constrained problems^11, 12^, error correcting codes^13, 14^, effective interaction among density fluctuations in heterogeneous frustrated glassy systems^15–20^ and fish shoals behavior^21, 22^ are significant diverse examples of direct problems in which nonlinearity plays a non-perturbative role in determining the system behavior. Nevertheless, to our knowledge, not many studies of the inverse problem have been performed so far in the field. In this work we aim at filling in this gap presenting a detailed analysis based on pseudolikelihood maximization (PLM) techniques for the statistical inference in models with multi-body interactions.

Inverse problems consist in determining the interaction couplings among system variables from measurements of variable configurations or correlations. As an instance, in the optical waves framework, this means quantitatively inferring the nonlinear interaction strengths given the wave emissions. Once the theoretical model is designed, assuming an effective equilibrium (true thermodynamic equilibrium or stationary conditions), one has to maximize the likelihood functional with respect to the coupling parameters. The likelihood functional is defined as the probability of a variable configuration given the values of the interaction couplings. For large systems it is numerically intractable but one can resort to the so-called pseudolikelihood functional defined as the probability of one variable conditional to all other variables and to the values of the couplings^23^.

Based on pseudolikelihood maximization, we adopt two methods to determine the interactions: the well known ${\ell }_{1}$-regularization^24, 25^, that we have improved with a hypothesis testing procedure based on the evaluation of the eigenvalues of the Fisher information matrix^26^, and the most recent decimation technique^27^. In order to test the methods, we considered both the phasor and the *XY*-spin models, generating the data by means of Monte Carlo numerical simulations. Among the simulated networks we analyze both sparse graphs, in which the number of interacting quadruplets *N*_*q*_ scales like the number of variables, *N*_*q*_ ∝ *N*, and dense graphs, in which *N*_*q*_ ∝ *N* ^3^ [This is a diluted dense graph: not all quadruplets are present, though their number per variable node scales with *N*, unlike in sparse graphs. A complete dense graph would contain *O*(*N* ^4^) interacting quadruplets]. We stress that the techniques here reported might be applied to any wave system with non-linear collective behavior, such as phase-locking, breathers and synchronization^28–31^, including the prototype Fermi-Pasta-Ulam model^32^. Further on, the methodology can be translated to simpler cases, e.g., discrete variables models like the *p*-clock model^33, 34^, in which rotators only take *p* discrete values. Properly modifying the mode interaction these *p*-clock models can, eventually, represent multi-body Potts models^35^, as well.

## Results

### Test models

Our first test model consists of *N* phasors *a*_*k*_ with a global constraint ${\sum }_{k=1}^{N}\,{|{a}_{k}|}^{2}={\rm{const}}\times N$, hereafter termed Spherical Model (SM), with Hamiltonian^36^

1 $$ {\mathcal H} =-\frac{1}{8}\sum _{jklm}^{{\rm{d}}.{\rm{i}}.}\,{J}_{jklm}\,{a}_{j}{a}_{k}^{\ast }{a}_{l}{a}_{m}^{\ast }+{\rm{c}}.{\rm{c}}.$$

The *a*_*k*_’s represent, e.g., the complex amplitudes of the normal modes expansion of the electromagnetic field^1^

2 $$\tilde{E}({\boldsymbol{r}},t)=\sum _{k}\,{a}_{k}(t){{\boldsymbol{E}}}_{k}({\boldsymbol{r}}){e}^{i{\omega }_{k}t}+{\rm{c}}.{\rm{c}}.$$

characterizing the light modes in the ***E***_*k*_(***r***) basis. The amplitude *a*_*k*_(*t*) is the slowly varying coefficient of the normal mode ***E***_*k*_ of frequency *ω*_*k*_ and varies on time scales much slower than ${\omega }_{k}^{-1}$. We adopt it as test model because it is the lowest order of nonlinearity satisfying time reversal symmetry of light, as occurring, e.g., in centrometric crystals with symmetric atomic potentials^37^. The laser transition can be represented as a phase transition in statistical mechanical theory. This turns out to be possible both in ordered multimode mode-locked lasers^3, 5, 6, 34, 38–40^ and in random lasers^10, 36, 41, 42^. Considering further orders of the interaction does not change the critical behavior and the onset of the lasing regime, nor the qualitative features of the laser in the high pumping regime. We stress that, simply in order to focus the presentation, also lower order interactions (pairwise and three body) are not considered here: the sum with superscript “d.i.” in Eq. (1) is intended solely over quadruplets with distinct indices.

According to multimode laser theory^1–3, 37, 43^ modes do interact nonlinearly if and only if their frequencies satisfy a frequency matching condition^5^, i.e., given any four modes *j*, *k*, *l*, *m* of typical line-width *γ*, their angular frequencies are such that

3 $$|{\omega }_{j}-{\omega }_{k}+{\omega }_{l}-{\omega }_{m}|\mathop{ < }\limits_{ \tilde {}}\gamma $$

at least in one permutation of their indices.

With equipartite magnitudes ($|{a}_{k}|\simeq 1$, $\forall \,k$) or with quenched ones (|*a*_*k*_(*t*)| = *A*_*k*_(0)) Eq. (1) for phasors reduces to the so-called *XY* model for rotators, ${a}_{j}={A}_{j}{e}^{\iota {\varphi }_{j}}\to {e}^{\iota {\varphi }_{j}}$, with Hamiltonian

4 $$\begin{array}{rcl} {\mathcal H}  & = & -\frac{1}{8}\sum _{jklm}^{{\rm{d}}.{\rm{i}}.}\,[{J}_{jklm}^{R}\,\cos ({\varphi }_{j}-{\varphi }_{k}+{\varphi }_{l}-{\varphi }_{m})\\  &  & +{J}_{jklm}^{I}\,\sin ({\varphi }_{j}-{\varphi }_{k}+{\varphi }_{l}-{\varphi }_{m})]\end{array}$$

where *J* ^*R*,*I*^ are, respectively, real and imaginary parts of the coupling constants. The 4*XY* model is our second test model. Besides being an approximation of the SM model, having locally constrained variables allows for testing the inference techniques also on sparse graphs at low temperature [Indeed, it can be seen that for bond-disordered SM’s, if the node connectivity does not increase at least with *N* ^2^, all the power condensates into one single quadruplet below threshold^6^]. Furthermore, terming *δω* the frequency spacing among the modes, we considered both strict frequency matching conditions, cf. Eq. (3), based on comb-like^44^ single mode resonance distributions ($\gamma \ll \delta \omega$), as well as *narrow*-*band* conditions (*γ* > *δω*). In the latter case Eq. (3) does not play any role and the node frequencies have no influence on the structure of the graphs. On the other hand, in graphs built considering $\gamma \ll \delta \omega$ frequencies do play an important role. These will be called Mode-Locked (ML) graphs.

We infer data within the Boltzmann-Gibbs equilibrium hypothesis (see Suppl. Mat. for data generation). Then, the probability of a configuration ***a***, given a set ***J***, i.e., the likelihood functional, reads:

5 $$P({\boldsymbol{a}}|{\boldsymbol{J}})=\frac{1}{Z[{\boldsymbol{J}}]}\,\exp \,\{-\beta  {\mathcal H} [{\boldsymbol{a}}|{\boldsymbol{J}}]\}$$

Computing *Z*[***J***] is very hard in general. To circumvent this bottleneck one first defines the single variable pseudo-likelihood^23^ of the values of *a*_*i*_ biased by all other ***a***_\*i*_ values, and by ***J*** (see details in Suppl. Mat.)

6 $${P}_{i}({a}_{i}|{{\boldsymbol{a}}}_{\backslash i},{\boldsymbol{J}})=\frac{1}{{Z}_{i}[{{\boldsymbol{a}}}_{\backslash i},{\boldsymbol{J}}]}\,\exp \,\{{a}_{i}{H}_{i}[{{\boldsymbol{a}}}_{\backslash i},{\boldsymbol{J}}]+{\rm{c}}.{\rm{c}}.\}$$

with

7 $${H}_{j}[{{\boldsymbol{a}}}_{\backslash j},{\boldsymbol{J}}]=\sum _{klm\ne j}^{{\rm{d}}.{\rm{i}}.}\,{J}_{jklm}{ {\mathcal F} }_{klm}$$

8 $${ {\mathcal F} }_{klm}=\frac{1}{3}[{a}_{k}^{\ast }{a}_{l}{a}_{m}^{\ast }+{a}_{k}{a}_{l}^{\ast }{a}_{m}^{\ast }+{a}_{k}^{\ast }{a}_{l}^{\ast }{a}_{m}]$$

9 $${Z}_{i}[{{\boldsymbol{a}}}_{\backslash i},{\boldsymbol{J}}]\equiv \sum _{{a}_{i}}\,\exp \,\{{a}_{i}{H}_{i}[{{\boldsymbol{a}}}_{\backslash i},{\boldsymbol{J}}]+{\rm{c}}.{\rm{c}}.\}$$

Further on, if one considers *M* independent configurations {***a*** ^(*μ*)^}, with *μ* = 1, *μ* = 1, …, *M*, the pseudolikelihood of all the single node variables $\{{a}_{i}^{(\mu )}\}$, given all the others $\{{{\boldsymbol{a}}}_{\backslash i}^{\mu }\}$ and the couplings ***J***, factorizes. In order to deal with sums instead of products, one usually evaluates the log-pseudolikelihood that, thus, reads

10 $$\begin{array}{rcl}{ {\mathcal L} }_{i}^{\mathrm{(0)}}(\{{{\boldsymbol{a}}}_{\backslash i}^{\mu }\},{\boldsymbol{J}}) & = & \sum _{\mu =1}^{M}({a}_{i}^{\mu }{H}_{i}[{{\boldsymbol{a}}}_{\backslash i}^{\mu },{\boldsymbol{J}}]+{\rm{c}}.{\rm{c}}.)\\  &  & -\sum _{\mu =1}^{M}\,\mathrm{ln}\,{Z}_{i}[{{\boldsymbol{a}}}_{\backslash i}^{\mu },{\boldsymbol{J}}]\end{array}$$

The ***J***’s maximizing ${ {\mathcal L} }_{i}^{\mathrm{(0)}}$s are considered as the most probable couplings that originate the {***a*** ^(*μ*)^} configurations.

We analyze data from systems whose coupling values are randomly generated with a bimodal distribution $P(J)=\mathrm{1/2}[\delta (J-\hat{J})+\delta (J+\hat{J})]$, where $\hat{J}=\mathrm{1/}{N}^{(z-\mathrm{1)/2}}$, when the total number of quadruplets scales as *N*_*q*_ ~ *N* ^*z*^. This is the case, e.g., of the frustrated glassy random lasers^10, 45–49^, but the methods here exposed also work for the simpler cases of uniform couplings, like in standard mode-locking lasers^2, 3, 5, 34^ and random couplings with a relative small fraction of negative values, e.g., random unfrustrated lasers^34, 42, 50^.

## Data Analysis

### Decimation and ℓ_1_-regularization

We compare inference predictions obtained by different implementations of PLM. The ${\ell }_{1}$-regularization consists in adding to Eq. (10) a regularizing term ${ {\mathcal L} }_{i}^{\mathrm{(0)}}-\lambda {\Vert J\Vert }_{1}$, penalizing large ***J*** values and it is known to be particularly useful in retrieving sparse systems^25^. The *decimation* procedure, instead, iteratively removes the smallest couplings (cf., Suppl. Mat.). In this procedure one maximizes the *total* log-pseudolikelihood, summed over all the modes, i.e.,

11 $$ {\mathcal L} ({\boldsymbol{J}})\equiv  {\mathcal L} =\frac{1}{N}\sum _{i}^{N}\,{ {\mathcal L} }_{i}^{\mathrm{(0)}}$$

It is important to underline that, by maximizing each ${ {\mathcal L} }_{i}^{\mathrm{(0)}}$ separately, cf. Eq. (10), each coupling *J*_*ijkl*_ turns out to be inferred four times with, generally, four different estimates. The mean value is, then, usually taken as best reconstructed value. By maximizing the total ${\mathcal L}$, instead, each coupling *J*_*ijkl*_ is inferred only once.

### Data size and external tuning

We consider the effects of varying the size *M* of data sets, as well as, the *temperature*-like parameter *T* that determines the strength of the interaction. *T* resembles real data noise^51^ or it is used to drive the system to a phase transition, if present. As it will be shown, functioning of PLM’s qualitatively change at criticality and in different thermodynamic phases.

### Quality indicators

To evaluate the performances of the techniques we will consider the following quality indicators: (i) the True Positive Rate (TPR), that is the fraction of true bonds also appearing in the inferred set of bonds, (ii) the True Negative Rate (TNR), that is the fraction of missing bonds also absent in the inferred set of bonds, and (iii) the reconstruction error

12 $${{\rm{err}}}_{J}\equiv \sqrt{\frac{{\sum }_{q}{({J}_{q}-{J}_{q}^{\ast })}^{2}}{{\sum }_{q}{J}_{q}^{2}}}$$

yielding how far the inferred values ${J}_{q}^{\ast }$ of the distinct quadruplets $q\equiv \{i,j,k,l\}$ are from the true values *J*_*q*_. Exclusively for the decimation PLM, in order to reconstruct the number of non-zero couplings, i.e., the number of quadruplets actually present in the system, we analyze also the behavior of the *tilted* pseudolikelihood function defined as:

13 $${ {\mathcal L} }_{t}\equiv  {\mathcal L} (x)-x{ {\mathcal L} }_{{\rm{\max }}}-(1-x){ {\mathcal L} }_{{\rm{\min }}}$$

where *x* is the number of non-decimated, i.e., non-erased, couplings. ${ {\mathcal L} }_{{\rm{\max }}}$ is the maximum of the total log-pseudolikelihood, Eq. (11), at the beginning of the decimation procedure, when all possible couplings are contemplated, while ${ {\mathcal L} }_{{\rm{\min }}}$ is evaluated on a graph with no links. ${\mathcal L} (x)$ is the maximum with respect to the *x* fraction of all possible couplings that are still considered to be important parameters of the problem. Erasing irrelevant couplings does not affect ${\mathcal L} (x)$ so that a plateau occurs in *x* > x* until important couplings start to be decimated and ${\mathcal L} (x)$ starts to decrease. In order to ease the identification of the optimal number of fitting parameters x*, ${\mathcal L} (x)$ is tilted: the optimal value x*, corresponding to the amount of couplings in the true network, is determined looking at the maximum of ${ {\mathcal L} }_{t}$.

**Figure 1:**
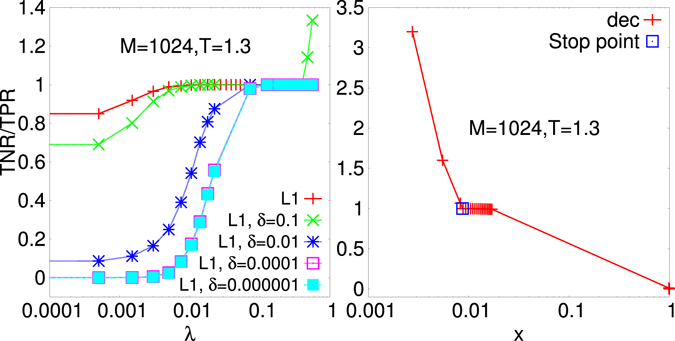
The TNR/TPR ratio vs. the regularizer *λ* used for the ${\ell }_{1}$-regularization (left) and vs. the fraction *x* of undecimated couplings for the PLM with decimation. The stopping point indicates the maximum of ${ {\mathcal L} }_{t}$, Eq. (13), where the decimation procedure stops. In the first case two different criteria are chosen to eliminate small bonds: the *a*-*priori δ* thresholding or the *a posteriori* inferred bond distribution thresholding based on the Fisher information matrix (see Suppl. Mat. for details). Data are taken from a 4*XY* model on a sparse Erdos-Renyi random graph with *N*_*q*_ = *N*, *N* = 16, *T* = 1.3, *M* = 1024. In this case the finite size proxy for the critical temperature is ${T}_{c}\mathrm{(16)}\simeq 1.34$.

In Fig. 1, using data from a 4*XY* model on Erdos-Renyi (ER)-like sparse graph, we show how the TNR/TPR ratio increases to 1 as the parameter *λ* used for the ${\ell }_{1}$-regularization, i.e., ${ {\mathcal L} }_{i}^{\mathrm{(0)}}-\lambda {\Vert J\Vert }_{1}$, is increased. Further on a *δ*-threshold criterion^25^ is adopted for *model selection*, i.e., the ability to reduce the number of parameters to the relevant ones. Within the *δ*-threshold criterion, couplings which are inferred, in absolute value, to be less than *δ* are considered to be irrelevant and are set to zero. The value for *δ* is, however, chosen *a priori* and the choice might be delicate when there is not a clear gap in the distribution of the inferred couplings^27^. Moreover, as *λ* is small, we see that the smaller the *δ* the less precise the network reconstruction. On the other hand, the smaller the *λ* the less perturbed the original pseudolikelihood (PL). Indeed, increasing *λ* the chance of globally underestimate the couplings increases.

**Figure 2:**
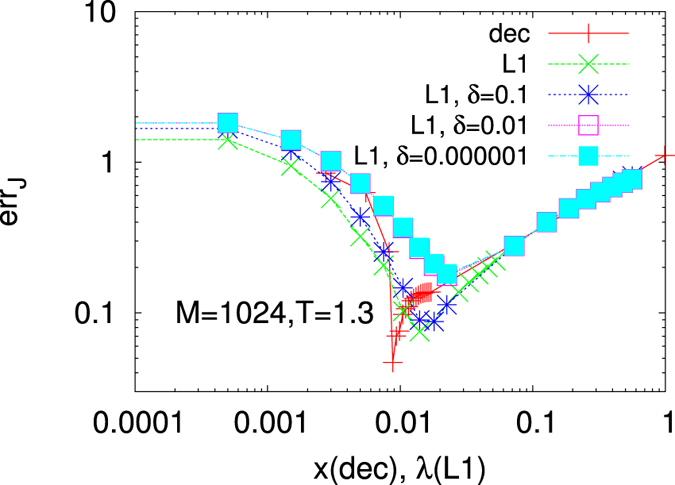
Reconstruction error for the 4*XY* model on sparse Erdos-Renyi graph with *N*_*q*_ = *N* = 16, *M* = 1024 at *T* = 1.3. The error obtained following various ${\ell }_{1}$-regularized PLMs is displayed vs. *λ*; the decimation PLM reconstruction error is plotted against the fraction of non-decimated couplings *x*.

If the probability distributions of the estimators are known, the issues related to an *a priori* fixing of a *δ* threshold might be overcome through a more accurate *hypothesis testing* procedure. Indeed, it can be seen that, as *M* → ∞, the probability distribution of the maximum PL estimator is a Gaussian with variance given by the diagonal element of the inverse of the Fisher information matrix^26^. Therefore, as detailed in Suppl. Mat., we can construct a confidence interval for each estimated value and verify whether it is compatible with the hypothesis “being a zero coupling”. If it is the case, it is considered as an irrelevant parameter and erased. As we can see from Figs 1 and 2 this criterion for model selection outperforms, for every value of *λ*, the *δ*-threshold method. Moreover, as detailed in the Methods, this criterion provides a method to determine the best value for the regularizer *λ*. *λ* is usually chosen through Cross-Validation (CV) or Generalized CV techniques (see, e.g., ref. 52). CV techniques are, however, much more computational demanding and the number of samples used to fit the model and infer the interaction couplings is further reduced in order to have a validation set (see Methods for more details on the application of the CV technique on this model inference). Always in Fig. 1 (right) we display the TNR/TPR ratio obtained with the decimation PLM as the fraction of non-decimated couplings *x* decreases (from fully connected limit *x* = 1 to non-interacting graph *x* = 0). At *x* = 1 the TPR is always one, for any *M* and *T*, whereas the TNR = 0. As the fraction of non-decimated couplings decreases but remains greater than or equal to the true one (x* = 2/15 in the original model analyzed in the right panel of Fig. 1) the TPR does not decrease and the TNR increases towards one. Eventually, more couplings than those of the original network are decimated: the TPR starts decreasing and the ratio TNR/TPR consequently grows above 1 as *x* → 0. The blue square indicates the stopping point of the decimation procedure determined, instead, as the maximum of the ${ {\mathcal L} }_{t}$, cf. Eq. (13). It can be observed that in this case it perfectly reconstructs the network of interactions since the TPR = TNR = 1.

### Decimation PLM

When comparing the performances of the most efficient regularization method with the decimation one, we observe that the network reconstruction in terms of true and false couplings is very adequate with both methods. However, the order of magnitude of the reconstruction error, testing also the quality of the inferred *values* of the couplings, is smaller in the decimation PLM, cf. Fig. 2, when the fraction of decimated couplings (1 − *x*) equals the one of the true network. It is important to underline that, within the decimation PLM, no parameters are determined *a priori*: the optimum value of *x* is determined maximizing the ${ {\mathcal L} }_{t}$. Moreover, exact fraction of relevant parameters and best estimate of their values are simultaneously inferred, which is not always true in the PLM with ${\ell }_{1}$-regularization since even the smallest *λ* for network reconstruction might induce a too high global underestimation of the couplings. We, thus, deepen the analysis of the decimation PLM.

**Figure 3:**
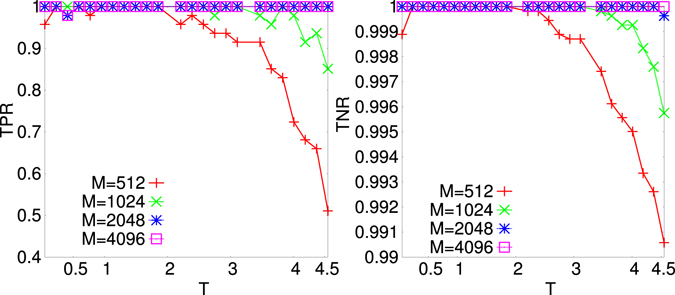
TPR (Left) and TNR (Right) for decimated networks at the maximum *x* ^*M*^ of the tilted pseudolikelihood ${ {\mathcal L} }_{t}$ vs *T* at different data-set sizes *M* for the 4*XY* model on sparse Mode-Locked graphs with *N* = 16, *N*_*q*_ = 47, ${T}_{c}(N)\simeq 0.50$ (*T*_*c*_(∞) = 0).

**Figure 4:**
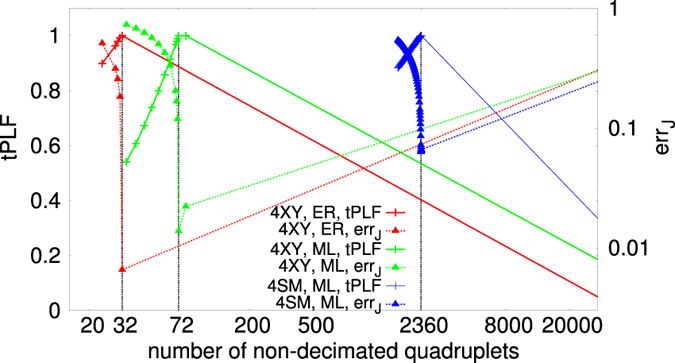
Tilted pseudolikelihood tPLF (normalized to its maximum) and reconstruction error for different models. Concave curves are tPLF, convex curves are err_*J*_. Three apart models on different random graphs are considered. Red (left) curves: 4*XY* model on sparse Erdos-Renyi graph with *N* = 32, *N*_*q*_ = 32, *M* = 65000, at *T* = 1.2 (${T}_{c}\mathrm{(32)}\simeq 1.39$). Green (mid) curves: 4*XY* model on sparse Mode-Locked graph with *N* = 32, *N*_*q*_ = 72, *M* = 65000, at *T* = 1.8 (${T}_{c}\mathrm{(32)}\simeq 0.72$). Blue (right) curves: 4SM model on dense Mode-Locked graph with *N* = 32, *N*_*q*_ = 2360, *M* = 65000, at *T* = 6.2 (${T}_{c}\mathrm{(32)}\simeq 0.91$).

**Figure 5:**
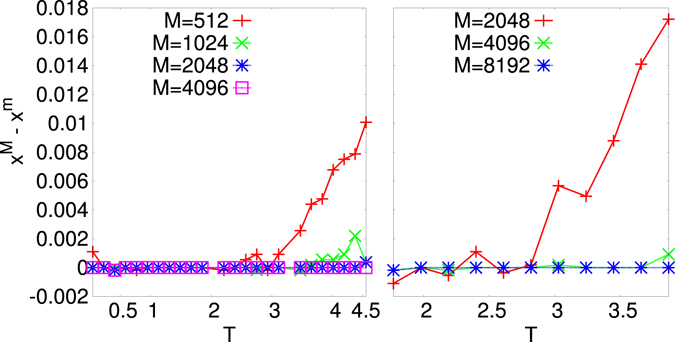
Plot of the difference between the maximum point of ${ {\mathcal L} }_{t}$, *x* ^M^, and the minimum point of the reconstruction error, *x* ^m^, vs *T* at different *M* for systems of *N* = 16 variables. Left: 4-*XY* model on sparse Mode-locked graph with *N*_*q*_ = 47 (${T}_{c}\mathrm{(16)}\simeq 0.50$). Right: 4SM model on dense Mode-Locked graph with *N*_*q*_ = 252 (${T}_{c}\simeq 1.07$).

**Figure 6:**
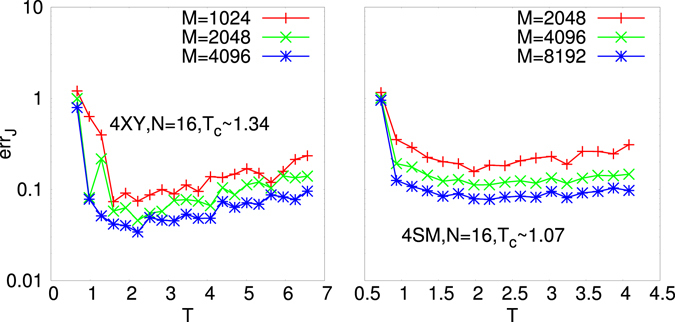
Reconstruction error at its minimum *x* ^min^ = *x* ^true^ in the decimation procedure vs. *T* for various *M* for *N* = 16 variables systems with bimodal random values of the coupling constants. Left: 4*XY* model on sparse Erdos-Renyi graph with *N* = 16, *N*_*q*_ = 16. Right: 4SM model on dense Mode-Locked graph with *N* = 16, *N*_*q*_ = 252.

In Fig. 3 we display TPR (left) and TNR (right) vs. *T* and *M* for the decimated network at the maximum point, *x* ^*M*^, of the ${ {\mathcal L} }_{t}$ for the 4*XY* model on a Mode-Locked-like sparse graph with *N*_*q*_ = 47 number of quadruplets and *N* = 16 nodes. For large enough *M* the reconstruction is optimal for all temperatures, whereas for small *M* it is guaranteed only in a *T* interval around the *finite size* proxy to the critical temperature (see Suppl. Mat.). Indeed, we observed that, tuning the external temperature-like parameter for each system studied, one can identify a “critical” *T* interval where the reconstruction error is minimal, even orders of magnitude smaller than outside such interval, and, more in general, the system is better and easier reconstructed. In Fig. 4, the behavior of the ${ {\mathcal L} }_{t}$ versus *x* is compared to err_*J*_(*x*) for three different systems, the 4*XY*-model on ML and ER sparse graphs and the 4SM-model on a dense ML graph. All systems have *N* = 32 variable nodes while the number of interaction quadruplets is *N*_*q*_ = 32, 72 and 2360, respectively. The number of configurations in all cases is *M* = 65000. It is clearly observed that, given a large enough *M* and/or a critical-like *T* the maximum point of the ${ {\mathcal L} }_{t}$, *x* ^*M*^, coincides with the minimum point of err_*J*_, *x* ^*m*^. The decimation PLM gives then a criterion to determine the number of interaction couplings in the system from measurements data without any *a priori* chosen parameters. We notice that, as *M* is small and *T* far from the critical region, the maximum point of ${ {\mathcal L} }_{t}$ and minimum point of err_*J*_ can be mismatched, as shown in Fig. 5. In Fig. 6 the *T* dependence of err_*J*_ is plotted for the 4*XY* model on a ER sparse graph and for the 4SM model on a ML dense graph at different values of *M*. As detailed in Suppl. Mat. the critical *T* interval turns out to be identified by the (finite size) critical temperature estimate of the phase transition point of the direct statistical mechanical problem.

## Discussion

We have been applying and improving Pseudo-Likelihood Maximization(PLM) techniques to the inverse problem in multi-body models, representing systems with nonlinerar response in generic theories. Firstly, we have quantitatively measured the quality of the network reconstruction showing that both PLM methods analyzed allow to obtain an optimal reconstruction for these systems in all thermodynamic phases, when large enough number of samples *M* are available. Decreasing *M* the optimal reconstruction is better achieved around the finite size proxy to the critical temperature. Performing then a deeper analysis of the reconstruction error, which gives information on how far the inferred couplings are from the true couplings, reveals that with the decimation PLM the inferred values are closer to those ones of the original systems. Our analysis has been motivated by the study of lasers in the framework of statistical mechanics, though, looking at the models employed, Eqs (1 and 4), its range of applicability is more widespread and potentially involves many problems in which both nonlinear and multi-body contributions turn out to be relevant in determining the system behavior, see, e.g., refs 2, 9, 12–14, 17, 21. Focusing on optics, data from experiments would allow to identify active and passive mode-locking in multimode lasers in terms of mode-coupling coefficients. When more modes on the network graph are connected by a non-vanishing coupling they are matched in frequency, cf. Eq. (3), and therefore, beyond some critical point, they will be locked in phase. Configurations of magnitudes and phases can be obtained from the Fourier analysis of the pulses in ultrafast multimode lasers^53–55^, pulses known to occur because of mode-locking, and parameters like the self amplitude modulation coefficients of saturable absorbers and the Kerr parameter can be inferred. In presence of relevant light scattering, instead, occurring in random lasers, no direct measurements of phases has been carried out so far, to our knowledge, but only spectral intensities, i.e., modes magnitudes. Acquiring also phases configurations, our inference technique would give the possibility to determine the strength of the interaction among the modes in the systems and to discriminate whether or not self-starting mode-locking occurs in random lasers.

## Electronic supplementary material

Supplemental Material for Inverse problem for multi-body interaction of nonlinear waves
