# Supplementary material for: Inverse problem for multi-body interaction of nonlinear waves
Source: Sci Rep. 2017 Jun 14;7:3463. doi: 10.1038/s41598-017-03163-4 (PMC5471250; doi:10.1038/s41598-017-03163-4)
Supplement: Supplementary file 1 — Supplemental Material for Inverse problem for multi-body interaction of nonlinear waves [file 41598_2017_3163_MOESM1_ESM.pdf]

Supplemental Material for *Inverse problem for multi-body interaction of nonlinear waves*

Alessia Marruzzo*[1], Payal Tyagi[1], Fabrizio Antenucci[1], Andrea Pagnani[2,3], and Luca Leuzzi[1,4]

[1]NANOTEC-CNR, Institute of Nanotechnology, Soft and Living Matter Laboratory, Rome, Piazzale A. Moro 2, I-00185, Roma, Italy
[2]Department of Applied Science and Technology, Politecnico di Torino, 10129, Torino, Italy
[3]Human Genetics Foundation, Molecular Biotechnology Center, 10126 Torino, Italy
[4]Dipartimento di Fisica, Università di Roma "Sapienza", Piazzale A. Moro 2, I-00185, Roma, Italy

March 26, 2017

Data Generation. Configurations are generated by means of Monte Carlo numerical simulations of the Hamiltonians of the SM, Eq. (1), and the XY, Eq. (4) models with number of modes $N = 8, 16, 32$. In the SM model diluted dense graphs are simulated, i. e., with a total number of quadruplets $N_q \sim N^3$ and with an average number of couplings per variable $N_q/N = O(N^2)$. We generate graphs of two kinds. One kind is *à la* Erdos-Renyi: every mode participates in a number of couplings chosen with a Poissonian distribution but independently from each other. The other kind is Mode-Locked: a mode interacts with other three modes only if their frequencies satisfy one of the frequency matching condition permutations, cf. Eq. (3). In the XY model graphs are sparse, i. e., $N_q/N = O(1)$ and we generate both models on standard ER graphs and on ML graphs. In order to speed up thermalization and have access to data at equilibrium the Parallel Tempering algorithm has been used. Configuration recording is performed at Monte Carlo steps far enough to avoid data correlation in the critical region.

Maximization algorithm. In our analysis, rather than maximizing $\mathcal{L}_i^{(0)}(\boldsymbol{J}) - \lambda||J||_1$ and $\mathcal{L}(x, \boldsymbol{J})$, we invert the functional signs and minimize, in order to use well established packages available. For the minimization of -PL with ℓ_1-regularization we adopted L1General [1]: a set of MATLAB routines implementing several of the available unconstrained minimization strategies for solving ℓ_1-regularization problems. In particular, the results here presented were obtained with the spectral projected gradient method [1]. For the minimization of $-\mathcal{L}(x, \boldsymbol{J})$ in the decimation procedure, we use, instead, Open Optimization Library (OOL) [2], which is a set of constrained optimization codes written in C. The results here presented were obtained using the spectral projected gradient method for convex set [3]. The decimation code using the OOL routine has been *ad hoc* developed with parallel programming on GPU's, reducing the running times of numerical processing with respect to C++ codes on CPU up to a factor 5 for the largest analyzed systems, $N = 32$. In terms of timing, the parallel CUDA code outperforms the MATLAB routines up to a factor 30 for $N = 16$.

Inverse algorithm To evaluate the inverse of the Fisher Information matrix, defined by

$$\mathcal{I}^i_{ab} = -\frac{\partial^2 \mathcal{L}_i}{\partial J_a \partial J_b}\Big|_{\hat{\mathbf{J}}} \qquad (1)$$

where with a, b we indicate two possible quadruplets node i might belong to (see next paragraph), we first evaluate its Cholesky decomposition, being $\mathcal{I}^i_{ab}$ a symmetric, positive define square matrix, and then its inverse through GSL libraries.

Pseudolikelihood function From Eq. (1) in the main text, the likelihood function of a mode amplitude configuration $\boldsymbol{a}$, given a coupling set $\boldsymbol{J}$, is readily introduced as

$$P(\boldsymbol{a}|\boldsymbol{J}) = \frac{1}{Z[\boldsymbol{J}]} \exp\{-\beta \mathcal{H}[\boldsymbol{a}|\boldsymbol{J}]\}$$

To construct the likelihood function of a_j, i. e., the probability distribution of a single variable given the values of all the other variables $\boldsymbol{a}_{\setminus j}$, we first rewrite Eq. (1) in an equivalent way:

$$\mathcal{H}[\boldsymbol{a}|\boldsymbol{J}] \quad = \quad -\frac{1}{8}\sum_{j=1}^{N} a_j H_j[\boldsymbol{a}_{\setminus j}, \boldsymbol{J}] + \text{c.c.}$$

*Correspondence to alessia.marruzzo@uniroma1.it

defining the complex-valued local effective fields

$$H_j[\boldsymbol{a}_{\backslash j}, \boldsymbol{J}] = \frac{1}{4}\sum_{klm\neq j}^{\rm d.i.} J_{jklm}\mathcal{F}_{klm}$$
$$\mathcal{F}_{klm} = \frac{1}{3}\left[a_k^* a_l a_m^* + a_k a_l^* a_m^* + a_k^* a_l^* a_m\right]$$

Then, we separate the contributions from a given variable a_i from all contributions not involving a_i, only $\boldsymbol{a}_{\backslash i}$:

$$\begin{aligned}\mathcal{H}[\boldsymbol{a}|\boldsymbol{J}] &= -\frac{1}{8}a_i H_i[\boldsymbol{a}_{\backslash i}, \boldsymbol{J}] \\ & -\frac{1}{8}\sum_{j\neq i}^{1,N} a_j H_j[\boldsymbol{a}_{\backslash j}, \boldsymbol{J}] + \text{c.c.} \\ &= \mathcal{H}_i[a_i|\boldsymbol{a}_{\backslash i}, \boldsymbol{J}] + \mathcal{H}_{\backslash i}[\boldsymbol{a}_{\backslash i}|\boldsymbol{J}] \end{aligned} \quad (2)$$

In terms of this decoupling the partition function reads:

$$Z = \sum_{\boldsymbol{a}_{\backslash i}} \exp\left\{-\beta \mathcal{H}_{\backslash i}[\boldsymbol{a}_{\backslash i}|\boldsymbol{J}]\right\} Z_i[\boldsymbol{a}_{\backslash i}, \boldsymbol{J}] \quad (3)$$

with

$$Z_i[\boldsymbol{a}_{\backslash i}] \equiv \sum_{a_i} \exp\left\{a_i H_i[\boldsymbol{a}_{\backslash i}, \boldsymbol{J}] + \text{c.c.}\right\},$$

i. e., Eq. (9) of the main text. In order to effectively carry out the sum over a_i values one has to recall that the phasors satisfy a global *spherical* constraint $\sum_j |a_j|^2 = \epsilon N$, with constant ϵ. Once all $\boldsymbol{a}_{\backslash i}$ are given, then, the value of $|a_i|$ is fixed,

$$|a_i| = \sqrt{\epsilon N - \sum_{j\backslash i} |a_j|^2}$$

and $\sum_{a_i}$ simply reduces to an integral on the angular phase variable $\phi_i \in [0 : 2\pi[$. Using Eqs. (2) and (3) of this section the pseudolikelihood function of the values of a_i, biased by $\boldsymbol{a}_{\backslash i}$ values, can be, eventually, written as

$$P_i(a_i|\boldsymbol{a}_{\backslash i}, \boldsymbol{J}) = \frac{1}{Z_i[\boldsymbol{a}_{\backslash i}, \boldsymbol{J}]} \exp\left\{a_i H_i[\boldsymbol{a}_{\backslash i}, \boldsymbol{J}] + \text{c.c.}\right\}$$

In order to find the best estimates of the interaction parameters given a data set of M mode amplitude configurations, we minimize the opposite log-pseudolikelihood functional $-\mathcal{L}_i$:

$$-\mathcal{L}_i = -\sum_{\mu=1}^{M}\left(a_i^{\mu} H_i[\boldsymbol{a}^{\mu}_{\backslash i}, \boldsymbol{J}] + \text{c.c.}\right) + \sum_{\mu=1}^{M} \ln Z_i[\boldsymbol{a}^{\mu}_{\backslash i}, \boldsymbol{J}] \quad (4)$$

with respect to the coupling parameters, exploiting the explicit knowledge of the derivatives

$$\frac{\partial(-\mathcal{L}_i)}{\partial J_{ijkl}} = \sum_{\mu=1}^{M} \mathcal{F}^{\mu}_{jkl}\left[\langle a_i\rangle_i^{\mu} - a_i^{\mu}\right] \quad (5)$$

where we denoted

$$\langle(\ldots)\rangle_i \equiv \frac{1}{Z_i[\boldsymbol{a}^{\mu}_{\backslash i}, \boldsymbol{J}]}\sum_{a_i}(\ldots)\exp\left\{a_i H_i[\boldsymbol{a}^{\mu}_{\backslash i}, \boldsymbol{J}] + \text{c.c.}\right\}.$$

Rewriting the complex amplitude in polar coordinates $a_i = A_i e^{\imath\phi_i}$ we have the following expression for the marginal (we do not write the $\boldsymbol{J}$ dependence)

$$\begin{aligned} P_i(A_i, \phi_i|\boldsymbol{A}_{\backslash i}, \boldsymbol{\phi}_{\backslash i}) &= \frac{\exp\left\{A_i\left[H_i^R\ \cos\phi_i + H_i^I\ \sin\phi_i\right]\right\}}{Z_i[\boldsymbol{A}_{\backslash i}, \boldsymbol{\phi}_{\backslash i}]} \\ &= \frac{\exp\left\{A_i|H_i|\cos(\phi_i - \gamma_i)\right\}}{2\pi\int dA_i\ I_0(A_i|H_i|)} \end{aligned} \quad (6)$$

where

$$|H_i| = \sqrt{\left(H_i^R\right)^2 + \left(H_i^I\right)^2}$$
$$\gamma_i = \arctan\frac{H_i^I}{H_i^R}$$

and $I_0(x)$ is the modified Bessel function of the first kind.

As mentioned in the main text, the polar coordinates are most useful in the cases of intensity equidistribution among the modes, $A_i \simeq 1$, $\forall\ i$, and of quenched amplitudes, i.e., when the A_i dynamics is quenched on the time scales of the ϕ's dynamics. In the latter case all A's are taken care of by rescaling the coupling constants as $A_i A_j A_k A_l J_{ijkl} \to J_{ijkl}$. When the couplings are considered real-valued, the polar expressions of the local effective fields, cf. Eq. (2) in the main text, can be rewritten by substituting Eq. (2) with

$$\begin{aligned}\mathcal{F}^R_{jkl} &= \cos\phi_j\cos\phi_k\cos\phi_l + \frac{1}{3}(\cos\phi_j\sin\phi_l\sin\phi_k \\ &+ \cos\phi_l\sin\phi_j\sin\phi_k + \cos\phi_k\sin\phi_j\sin\phi_l) \\ \mathcal{F}^I_{jkl} &= \sin\phi_j\sin\phi_k\sin\phi_l + \frac{1}{3}(\sin\phi_j\cos\phi_l\cos\phi_k \\ &+ \sin\phi_l\cos\phi_j\cos\phi_k + \sin\phi_k\cos\phi_j\cos\phi_l)\end{aligned}$$

and the log-pseudolikelihood functional $\mathcal{L}_i$ and its gradient, cf. Eqs. (4,5) in this section, simplify to

$$-\mathcal{L}_i = \sum_{\mu=1}^{M}\left\{\ln 2\pi I_0\left(\left|H_i(\boldsymbol{\phi}^{\mu}_{\backslash i})\right|\right) - \left[H_i^R(\boldsymbol{\phi}^{\mu}_{\backslash i})\cos\phi_i^{\mu} + H_i^I(\boldsymbol{\phi}^{\mu}_{\backslash i})\sin\phi_i^{\mu}\right]\right\} \quad (7)$$

$$\frac{\partial(-\mathcal{L}_i)}{\partial J_{ijkl}} = \sum_{\mu=1}^{M}\left\{\mathcal{F}^{\mu}_{jkl} \times \left[\frac{I_1(|H_i(\boldsymbol{\phi}^{\mu}_{\backslash i})|)}{I_0(|H_i(\boldsymbol{\phi}^{\mu}_{\backslash i})|)}\frac{H_i(\boldsymbol{\phi}^{\mu}_{\backslash i})}{|H_i(\boldsymbol{\phi}^{\mu}_{\backslash i})|} - e^{\imath\phi_i^{\mu}}\right] + \text{c.c.}\right\}$$

To determine the interaction network of non-linear wave systems we use and compare two techniques: the ℓ_1-regularization PLM [4, 5] and the decimation PLM [6].

ℓ_1-regularization In this approach we add a ℓ_1 norm contribution for each coupling to be inferred to the log-pseudolikelihood in order to keep the coupling values from diverging during the minimization procedure:

$$-\mathcal{L}_i \to -\mathcal{L}_i + \lambda \sum_{jklm}^{\text{d.i.}} |J_{jklm}| \tag{8}$$

The positive regularizer λ must be small in order to prevent the modification of the landscape of $\mathcal{L}_i$. We take a pseudolikelihood that is intensive in M but small enough values of λ for all M's.

Within this method, for each mode i all couplings involving i are inferred in one apart iteration. The same quadruplet J_{ijkl} can, thus, be inferred more times, proceeding by minimizing likelihood functions for different, though interacting, modes i, j, k and l. Nothing prevents the values of apart reconstructions to be the same. Eventually, thus, all inferred values of the same coupling are averaged in order to enforce the original symmetry.

δ thresholding A further improvement in the reconstruction of the topology can be achieved, as suggested in Ref. [5], by setting to zero all couplings whose estimate is below a threshold value δ. However, the choice of the δ value might be delicate since there are many cases in which there is no clear gap between the zero and the non-zero couplings [6]. If the probability distributions of the estimators are known, we can overcome this problem developing a more accurate hypothesis testing scheme.

$\mathcal{P}(\hat{J})$ thresholding It can be seen that, as $M \to \infty$, the probability distribution $\mathcal{P}(\hat{J})$ of the maximum PL estimator $\hat{J}$ converges to a Gaussian distribution centered around the true value of the coupling and with variance estimated by the diagonal elements of the inverse of the Fisher information matrix [7]. The elements, $\mathcal{I}^i_{ab}$, of the Fisher information matrix are defined through:

$$\mathcal{I}^i_{ab} = -\frac{\partial^2 \mathcal{L}_i}{\partial J_a \partial J_b}\bigg|_{\hat{\mathbf{J}}}$$

where with a, b we indicate two possible quadruplets including node i, i.e., $a = \{i,j,k,l\}$, $b = \{i,j',k',l'\}$. Then, knowing the distribution, we can determine, for every estimated value J^*_a, if it is compatible with a Gaussian centered in zero, i.e., if the hypothesis for the true coupling to be zero might or not be rejected.

The hypothesis testing can be schematically developed as follows.

(i) Once the maximum points of the PL are found, we evaluate the inverse of the Fisher information matrix, Eq. (9); from Eq. (7), we have:

$$\begin{aligned}\mathcal{I}^i_{ab} &= \sum_{\mu=1}^{M} \mathcal{F}^\mu_a \mathcal{F}^\mu_b \Bigg\{ \\ &\times \left(\frac{H_i(\phi^\mu_{\setminus i})}{\left|H_i(\phi^\mu_{\setminus i})\right|}\right)^2 \mathcal{B}\left(\left|H_i(\phi^\mu_{\setminus i})\right|\right) \\ &+ \frac{I_1(|H_i(\phi^\mu_{\setminus i})|)}{I_0(|H_i(\phi^\mu_{\setminus i})|)} \left(\frac{\left|H_i(\phi^\mu_{\setminus i})\right|^2 - \left(H_i(\phi^\mu_{\setminus i})\right)^2}{\left|H_i(\phi^\mu_{\setminus i})\right|^3}\right)\Bigg\}\end{aligned} \tag{9}$$

where with $\mathcal{B}(x)$ we indicate:

$$\mathcal{B}(x) = \frac{1}{2}\left(\frac{I_2(x)}{I_0(x)} + 1\right) - \left(\frac{I_1(x)}{I_0(x)}\right)^2$$

The diagonal terms of the inverse matrix are taken as estimates for the variances, σ^*_a, of the estimator distributions.

(ii) We assume every couplings to be zero; hence, we expect every estimated value J^*_a to be compatible with the $N(0, \sigma^*_a)$ distribution. We, then, construct a confidence interval C_n that should contain the estimated value J^*_a within a 97.5% probability.

(iii) If the inferred J^*_a is contained in C_n we accept the zero hypothesis and the coupling is set to zero.

Optimal λ regularizer It is clear from Eq. (8), that as λ increases it increases the tendency to globally underestimate the interaction couplings. Within the hypothesis testing procedure described more and more couplings become compatible with a Gaussian centered in zero and are considered as irrelevant. Indeed, as noticed in Fig. 1, $\text{err}_J(\lambda)$ increases above some optimal value $\hat{\lambda}$ that should depend on the number of configurations M available and on T. One procedure that might be adopted to determine $\hat{\lambda}$ consists in testing the PLM with ℓ_1-regularization on data relative to a known system and use the optimum value found to solve the inverse problem of new systems. However, there might be cases in which there are no solved inverse problems available and, in general, $\hat{\lambda}$ could depend on the details of the problem.

Cross-Validation (CV) techniques are often applied to determine the best value of λ on supervised learning algorithms. On this problem of model inference, we can also think of implementing a CV method with the following scheme: the observed configurations are divided in two sets, a training and a validating set; the training set is then used to fit the model, i.e., determine the interaction couplings as a function of the trial value for the regularizer; we then run a Monte-Carlo, with these inferred couplings, and look at equilibrium configurations from which

we extract four point correlations functions, $C^{\rm mc}_{ijkl}$; these correlations are then compared with those ones obtained from the configurations of the validating set, $C^{\rm val}_{ijkl}$; the optimal λ is taken as the value that minimize the distance among $C^{\rm mc}_{ijkl}$ and $C^{\rm val}_{ijkl}$.
The hypothesis testing procedure explained in the previous paragraph could also be considered as a tool to determine $\hat{\lambda}$. Indeed, within the PLM, every coupling J_{ijkl} has four estimators J^*_a and we evaluate the σ^*_a for each one. It might be that not all the four estimated values have the same compatibility with a Gaussian centered in zero since, due to the lack of configurations, one value might be overestimated. Increasing λ, the number of quadruplets for which the hypothesis test does not give the same answer for every estimated value decreases. We observed that the minimum λ value for which there are no quadruplets that answer differently to the hypothesis test is very close, within less than an 8% difference, to $\hat{\lambda}$.

PLM Decimation In the decimation procedure we maximize the total log-likelihood for all modes

$$\mathcal{L} \equiv \frac{1}{N}\sum_i^N \mathcal{L}_i^{(0)} \qquad (10)$$

starting from $\mathcal{L}_{\max}$ defined on a full graph and inferring all the values of the $N_q = N(N-1)(N-2)(N-3)/24 \sim N^4$ couplings on that network. Sorting the couplings by their absolute value and taking away (*decimating*) the N_0 smallest we are left with a network of $N_q - N_0 = xN_q$ non-zero couplings. A new inference iteration, including minimization of $\mathcal{L}(x)$ and sorting, will allow to decimate further another group of the smallest couplings. And so on and so forth, as far as the couplings of the decimated model network are more than the couplings of the true system. Indeed, as far as the number of inferred parameters is larger than the true number of parameters the pseudolikelihood is not expected to change and will stick to its maximum value $\mathcal{L}_{\max}$ (overfitting). The indication of the true number of couplings will be given, indeed, right by the x value of the fraction of non-decimated couplings across which $\mathcal{L}$ starts decreasing because the number of inferred parameters is less than the real number of parameters (underfitting). The minimum conceivable $\mathcal{L}(x)$ is for the non-interacting system: $\mathcal{L}_{\min} = \mathcal{L}(0)$. To enhance the determination of the true network connectivity x^*, below which statistical interpolation becomes less reliable, Decelle and Ricci-Tersenghi [6] introduced the *tilted* pseudolikelihood $\mathcal{L}_t$

$$\mathcal{L}_t \equiv \mathcal{L}(x) - x\mathcal{L}_{\max} - (1-x)\mathcal{L}_{\min}$$

such that

$$\mathcal{L}_t(0) = \mathcal{L}_t(1) = 0$$
$$\mathcal{L}_t(x \le x^*) \simeq x(\mathcal{L}_{\max} - \mathcal{L}_{\min}).$$

N	phasor ER	phasor ML	XY ER	XY ML
16	$N_q = 366$	$N_q = 252$	$N_q = 16$	$N_q = 47$
32	$N_q = 2949$	$N_q = 2360$	$N_q = 32$	$N_q = 72$
128	$N_q = 188742$	$N_q = 168672$	$N_q = 128$	$N_q = 275$

Table 1: Total number of quadruplets in all simulated networks used to yield data to be tested by our pseudo-likelihood inference methods.

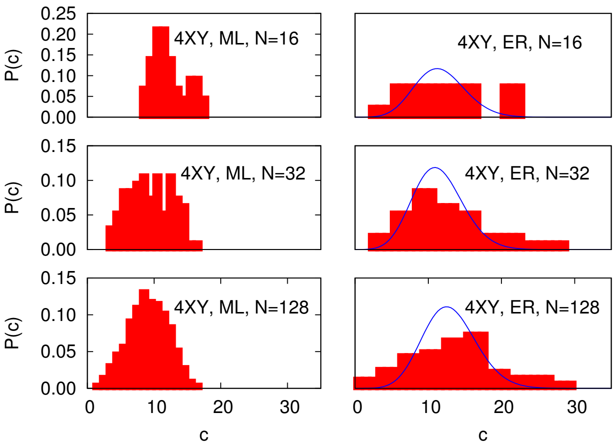

Figure 1: Distribution of connectivity per variable node of the 4XY models on ER-like (right) and Mode-Locking-like (left) sparse graphs of sizes $N = 16, 32, 128$. The blue curves in the right panels are Poisson distributions with same mean as the empirical distributions.

We stress that using the total likelihood, cf. Eq. (10), the symmetry of coupling constants under indices permutation is automatically enforced.

Random Graphs We report the distributions of connectivities in the various graphs that we used to test the reconstruction methods exposed in the main manuscript for the complex spherical model (SM), cf. Eq. (1), and the XY model, cf. Eq. (4), at finite N. In the XY models the x axis is the connectivity per variable. In the SM the total number of couplings scales like N^3: the x axis represents, in this case, the connectivity per variable node rescaled by N^2.

The distributions in the Erdos-Renyi-like graphs and in the Mode-Locked-like graphs in pairwise interacing systems are known to tend to Poissonian distributions in the thermodynamic limiti $N \to \infty$ [8], though the ML-graph convergence is much slower for increasing N. As a comparison, in the right panels of Figs. 1, 2 we plot the relative Poissonian distributions with the same average. Finite size effects are clearly strong for the small simulated sizes. The actual number of quadruplet couplings N_q for each simulated instance is reported for all consid-

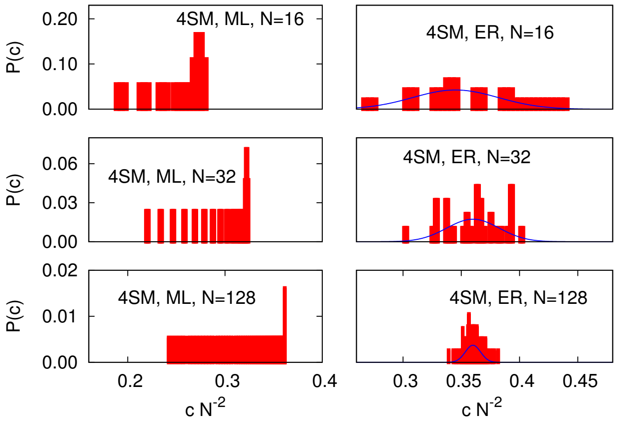

Figure 2: Distribution of rescaled connectivity c/N^2 in the 4-phasor models - termed "spherical model" (SM) in the plots - on ER-like and ML-like dense ($O(N^3)$) graphs of sizes $N = 16, 32, 128$. The blue curves in the right panels are Poisson distributions with same mean as the data derived distributions.

ered models in Tab. 1.

Finite size critical temperature We refer several times to a critical region in T for which inference works better than at higher or lower T. In particular, the reconstruction error is up to one order of magnitude smaller and the network topology is correctly reconstructed with the need of less configurations data. In Fig. 3 we display the energy plots of the models used to produce equilibrium data with Exchange Monte Carlo simulations. In the thermodynamic limit $N \to \infty$ they should display a true critical point behavior where a phase transition occurs from a paramagnetic-like phase to a phase-locked or to a spin-glass phase. For the 4XY model on ER sparse graphs these temperatures are analytically known, as $T_c = 0$ for $N_q/N = 1$ and $T_c > 0$ for $N_q/N = 2$ [9]. For finite sizes the mathematical discontinuity is approximated by a steep, though smeared, descent as T decreases. This step becomes steeper and steeper as N increases, eventually reaching the limit of a true singularity representing a true phase transition. Indeed, for finite systems, no actual phase transition occurs. However, one can identify finite size pseudocritical points that, for systems of increasing size N, form a series converging to the true critical point. Operatively, we take as finite size critical points $T_c(N)$ the peak of the specific heat dE/dT from $E_N(T)$ data in Fig. 3. For the simulated systems whose energy is displayed in Fig. 3 the $T_c(N)$ are reported in Tab. 2.

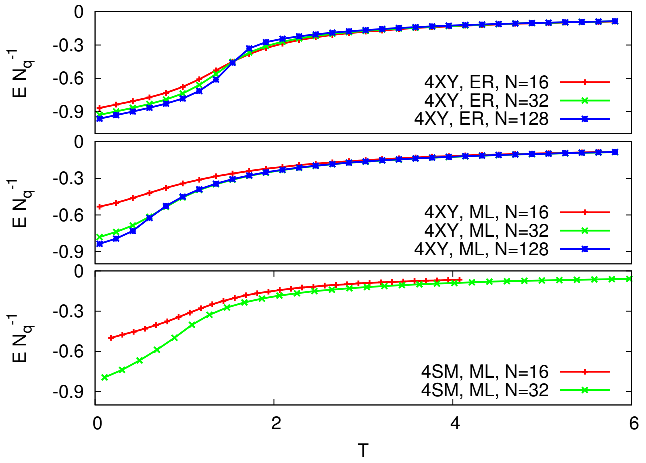

Figure 3: Internal energy vs. temperature in various simulated instances of the models of Eqs. (1,4)

N	phasor ML	XY ER	XY ML
16	1.07(1)	1.34(1)	0.50(1)
32	0.91(1)	1.39(1)	0.72(1)
128		1.53(1)	0.63(1)

Table 2: Finite size proxies for the critical point.

References

1. Schmidt, M. *Graphical Model Structure Learning with L1-Regularization* PhD thesis (University of British Columbia, 2010).

2. Biloti, R., D'Afonseca, L. & Ventura, S. *Open Optimization Library* <`http://ool.sourceforge.net/`> (2005).

3. Birgin, E. G., Martínez, J. M. & Raydan, M. Nonmonotone Spectral Projected Gradient Methods on Convex Sets. *SIAM Journal on Optimization* **10,** 1196–1211 (2000).

4. Ravikumar, P., Wainwright, M. J. & Lafferty, J. D. High-dimensional Ising model selection using ℓ_1 regularized logistic regression. *Ann. Statist.* **38,** 1287–1319 (2010).

5. Aurell, E. & Ekeberg, M. Inverse Ising Inference Using All the Data. *Phys. Rev. Lett.* **108,** 090201 (2012).

6. Decelle, A. & Ricci-Tersenghi, F. Pseudolikelihood Decimation Algorithm Improving the Inference of the Interaction Network in a General Class of Ising Models. *Phys. Rev. Lett.* **112,** 070603 (2014).

7. Wasserman, L. *All of Statistics: A concise course in statistical inference* (Springer, New York, 2003).

8. Marruzzo, A. *Statistical Mechanics of continuous spin models and applications to nonlinear optics in disordered media,* PhD Thesis (Sapienza University of Rome, 2016).

9. Marruzzo, A. & Leuzzi, L. Multi-body quenched disordered XY and p-clock models on random graphs. *Phys. Rev. B* **93,** 094206 (2016).
